# Reconstruction of the microalga Nannochloropsis salina genome-scale metabolic model with applications to lipid production

**DOI:** 10.1186/s12918-017-0441-1

**Published:** 2017-07-04

**Authors:** Nicolás Loira, Sebastian Mendoza, María Paz Cortés, Natalia Rojas, Dante Travisany, Alex Di Genova, Natalia Gajardo, Nicole Ehrenfeld, Alejandro Maass

**Affiliations:** 1 Mathomics, Center for Mathematical Modeling, Universidad de Chile, Beauchef 851, 7th Floor, Santiago, Chile; 2 Center for Genome Regulation (Fondap 15090007), Universidad de Chile, Blanco Encalada 2085, Santiago, Chile; 3 Centro de Investigación Austral Biotech, Universidad Santo Tomás, Avenida Ejercito 146, Santiago, Chile; 4 Universidad Adolfo Ibáñez, Diagonal Las Torres 2640, Santiago, Chile

**Keywords:** Genome-scale Metabolic model, Nannochloropsis salina, TAG, Microalgæ

## Abstract

**Background:**

*Nannochloropsis salina* (= Eustigmatophyceae) is a marine microalga which has become a biotechnological target because of its high capacity to produce polyunsaturated fatty acids and triacylglycerols. It has been used as a source of biofuel, pigments and food supplements, like Omega 3. Only some *Nannochloropsis* species have been sequenced, but none of them benefit from a genome-scale metabolic model (GSMM), able to predict its metabolic capabilities.

**Results:**

We present iNS934, the first GSMM for *N. salina*, including 2345 reactions, 934 genes and an exhaustive description of lipid and nitrogen metabolism. iNS934 has a 90% of accuracy when making simple growth/no-growth predictions and has a 15% error rate in predicting growth rates in different experimental conditions. Moreover, iNS934 allowed us to propose 82 different knockout strategies for strain optimization of triacylglycerols.

**Conclusions:**

iNS934 provides a powerful tool for metabolic improvement, allowing predictions and simulations of *N. salina* metabolism under different media and genetic conditions. It also provides a systemic view of *N. salina* metabolism, potentially guiding research and providing context to *-omics* data.

**Electronic supplementary material:**

The online version of this article (doi:10.1186/s12918-017-0441-1) contains supplementary material, which is available to authorized users.

## Background

In the last few years, interest in microalgæ has risen because of their ability to produce a wide range of compounds, such as carotenoids [1–3], lipids [4–6], hydrogen [7, 8], proteins [9, 10] and starch [11]. These algal compounds have numerous relevant applications, from fine natural chemicals to biofuels and food additives. However, it is still a challenge to optimize algal biomass and specific lipid composition to reach an economically feasible bulk production of these compounds [12]. Understanding the complexity of algal metabolism is key to tackling this problem.

Metabolic networks provide an efficient framework to describe cellular metabolism and have become an important tool in metabolic engineering, facilitating strain optimization and reducing the need for expensive in vivo experiments [13]. In addition, metabolic models integrated with *omics* data, such as transcriptional profiling, allows development of a meaningful systemic representation of metabolism [14]. Genome-scale metabolic network models (GSMMs) have been successfully reconstructed for several model species and a few biotechnologically relevant organisms like *Escherichia coli* [15, 16], *Saccharomyces cerevisiae* [17] and *Arabidopsis thaliana* [18].

Several efforts have been made to model algæ metabolism [19]. Green algæ have received special attention, with eight metabolic models for *Chlamydomonas reinhardtii* [20–27], one for *Botryococcus braunii* [28], five for the genera Chlorella [29–33] and two for *Ostreococcus* [34]. Additionally, seven models for the diatom *Phaeodactylum tricornutum* [35–41], one for the multicellular brown algæ *Ectocarpus siliculosus* [42] and one for the coccolithophore *Emiliania huxleyi* [43]. One important alga that is absent in this list is the marine specie *Nannochloropsis salina*. Nannochloropsis species has emerged as a leading microorganism for biodiesel production, due to their high photoautotrophic biomass accumulation rates [44] and high lipid content [45], either in open ponds or enclosed systems. Additionally, successful cultivation of *Nannochloropsis* species on a large scale using natural sunlight has been achieved by several companies [46]. Moreover, *Nannochloropsis* has gained great interest because of its potential for bio-production of eicosapentaenoic acid (EPA) which is a relevant additive for human health [47–49] and nutrition [50–52]. EPA is one of the major fatty acids produced by *Nannochloropsis*. Indeed, it could represent over 30% of total fatty acid content under heterotrophic conditions [53] and over 28% under autotrophic conditions [54] in *Nannochloropsis*.

We present here iNS934, the first genome-scale functional metabolic model for *N. salina*, built with a strategy that integrates metabolic knowledge from several related species, genomic and transcriptomic data. In particular, we generated transcriptomic data for *N. salina* which allowed us to confirm coding sequences (CDS) in its genome and also discover new ones. iNS934 provides a detailed description of biosynthesis of lipids for the *Nannochloropsis* genus. Specifically, it describes reactions for the biosynthesis of polyunsaturated fatty acids such as EPA, arachidonic acid (ARA) and eicosatetraenoic acid (ETA). iNS934 was validated both qualitative and quantitatively, with an average error of 15% in the latter. Moreover, the model was used to propose knockouts that could improve the production of triacylglycerols (TAGs).

## Methods

### *N. salina* transcriptome

**Setting up culture conditions for RNA extraction** *N. salina* cells were obtained from Commonwealth Scientific and Industrial Research Organization (CSIRO) and identified as CS-190 *Nannochloropsis salina* CCAP 849/2. They were cultured in Artificial Sea Water (ASW), supplemented with f/2 medium [55] at 20 °C, with an illumination of white-blue leds (30 *μ*E photons m-2s-1) on a 24 h light/day cycle, primary in batch cultures, as described by Chen et al. [56]. For mRNA extraction, *N. salina* cells were collected at the exponential growth phase (~ 5×10^6^cell/mL) at the following conditions: (1) Dark, Low CO_2_ (DLC): 4 h dark with 1 L/min air influx (CO_2_ 0.03%), (2) High light, Low CO_2_ (HLLC): 2 h high light (1000*μ*E photons m-2s-1) and 1 L/min air influx (0.03% CO_2_) and (3) High light, High CO_2_ (HLHC): 2 h high light (1000*μ*E photons m-2s-1) and 1 L/min air influx (1.5% CO_2_). Total RNA was extracted from frozen cells, which were ground using a mortar and pestle, using TRIzol RNA Isolation Reagents (Invitrogen) according to the manufacturer. Total RNA and mRNA integrity were analyzed by running them on agarose gel and in an Agilent Bioanalyzer to evaluate its quality before sending it to library construction.

**EST collection/library construction and sequencing**

To obtain a good coverage of the *N. salina* transcriptome, two different sequencing techniques were adopted: GS FLX+ System (Roche), sequencing a normalized cDNA library, and Illumina sequencing a cDNA library. Regular and normalized library construction and sequencing was performed by Eurofin MWG Operon, USA.

For the Roche GS FLX sequencing, we made a RNA pool, including all 3 conditions previously described (DLC; HLLC; HLHC). To build the normalized cDNA library construction, from a total RNA sample, poly(A)+ RNA was isolated and used for cDNA synthesis. The poly(A)+ was fragmented by ultrasound (1 pulse of 30 s at 4 °C). First-strand cDNA synthesis was primed with a N6 randomized primer. Then 454 adapters A and B were ligated to the 5’ and 3’ ends of the cDNA. The cDNA was finally amplified with 13 PCR cycles using a proof reading enzyme. Normalization was carried out by one cycle of denaturation and reassociation of the cDNA, resulting in N1-cDNA. Reassociated ds-cDNA was separated from the remaining ss-cDNA (normalized cDNA) by passing the mixture over a hydroxylapatite column. After hydroxylapatite chromatography, the ss-cDNA was amplified with 14 PCR cycles. For Titanium sequencing the cDNA in the size range of 500–700 bp was eluted from a preparative agarose gel. Half a plate of GS FLX+ System (Roche) was sequenced.

Library preparation of total RNA from conditions HLLC and HLHC was carried out using the Illumina TruSeq kit. Cluster formation and sequencing on HiSeq2000 were done according to the manufacturer’s instructions. Two samples, one from each condition, were prepared in 250 bp paired-end sequenced in 2 different lanes, delivering around 40 million reads per lane.

***De novo*** **transcriptome assembly**

We divided this process in 4 steps: (1) Illumina raw data was error corrected, and then sequences were assembled using the Trinity package. (2) Roche 454 GS FLX raw reads were cleaned and trimmed with Figaro [57]. (3) Using BLASTN, 454 reads were mapped to Illumina contigs in order to avoid redundancy between corrected Illumina contigs and 454 data. (4) We generated 3 sets of data: The first was constructed using all Illumina transcripts without mapped 454 reads; the second set of data was constructed using 454 reads that had a hit against Illumina contigs and the corresponding Illumina reads. This data was reassembled using the Phrap^1^ software. The third dataset corresponds to the transcripts assembled by wgs-assembler, using 454 reads that did not have hits against Illumina transcripts. Transcripts from these three sets of data with less than 30 mapping reads or length under 300 bp were discarded. The remaining transcripts constitute our *de novo* transcriptome.

**Mapping transcriptome to reference genome**

We mapped this *de novo* transcriptome assembly to the *N. salina* CCMP537 genome assembly [58] (NCBI BioProject ID: PRJNA62503) using GMAP. Transcripts aligned to reference gene models with a coverage of at least 70% and and identity of 95% or higher were assigned to those genes. We identified coding regions on the remaining transcripts using TransDecoder^2^.

Functional annotation of coding regions from the reference genome and the supplementary transcripts from the *de novo* transcriptome was performed using BLAST searches (with an e-value threshold of 1e-10 and keeping the best ten hits) against Swissprot, KEGG, PRIAM and NR protein databases. Moreover, InterProScan (default parameters) was used to identify protein domains and GO numbers. In order to build a consensus annotation for each gene, the Gene name (Swissprot, KEGG), EC number (KEGG, PRIAM), KO number (KEGG), GO number (Interpro), InterPro number (Interpro) and protein product (Swissprot, KEGG, NR) attributes were obtained from BLAST and Interpro results; using an in-house PERL script; In parenthesis we show the databases from which attributes were obtained. Afterwards, a single value for each attribute was defined by picking the most frequent from the set of 10 best hits times the number of databases the attribute was parsed (i.e EC numbers can be obtained from KEGG and PRIAM results; then, we can count the most frequent from a list of 20 possible values). One exception to the previous rule was the protein product attribute; we chose it prioritizing the databases result in the following decreasing priority order: Swissprot, KEGG and NR.

The complete set of annotated CDS is included as Additional file 1.

### Reconstruction of *N. salina* metabolic model

The reconstruction of *N. salina* metabolic network was generated following the five stages described in the protocol for generating high-quality GSMMs [59]. We used as a reference *Chlamydomonas reinhardtii* and its iRC1080 metabolic model [22] in addition to *N. salina* genome annotation and literature. First, we searched for orthologs between our *N. salina* CDS and the *C. reinhardtii* protein sequences, using Inparanoid [60] and OrthoMCL [61]. Then we built a draft model for *N. salina* using *Pantograph* [62], taking as a template the iRC1080 model for *C. reinhardtii*. Then, we looked at the list of genes not present in the draft model, but which annotation included an Enzyme Commission (EC) number and we mapped those ECs to BiGG reaction identifiers. We imported those BiGG reactions into our model, using the BiGG web API^3^. Afterwards, we manually determined a list of reactions using different resources of information such as BIGG, MetaCyc and KEGG. These reactions were added to our reconstruction with their corresponding identifiers and *N. salina* gene associations. For reactions and metabolites without BiGG identifiers, BiGG-like identifiers were assigned. After adding reactions, we manually curated compartments, changed reversibility for some reactions, moved species among compartments, renamed and pruned unused elements, among other changes. In order to generate a functional model, we used meneco [63, 64] to look for BiGG reactions that could fill gaps in the model. meneco provided us with candidate reactions that were handed to the manual curators, who approved their inclusion into the model. We took metabolites available in the media as sources for gap-filling and the requirements for biomass as targets.

Formulas and charges for metabolites in our model were revised and all reactions in our model were subjected to mass and charge balances. Except light and exchange reactions, all reactions in our model are mass balanced and only 6% could not be charge balanced. The complete list of metabolites in the model with their formula, charge, BiGG ID and ID from the external database MetaNetX [65] is included in Additional file 2.

**Fig. 1:**
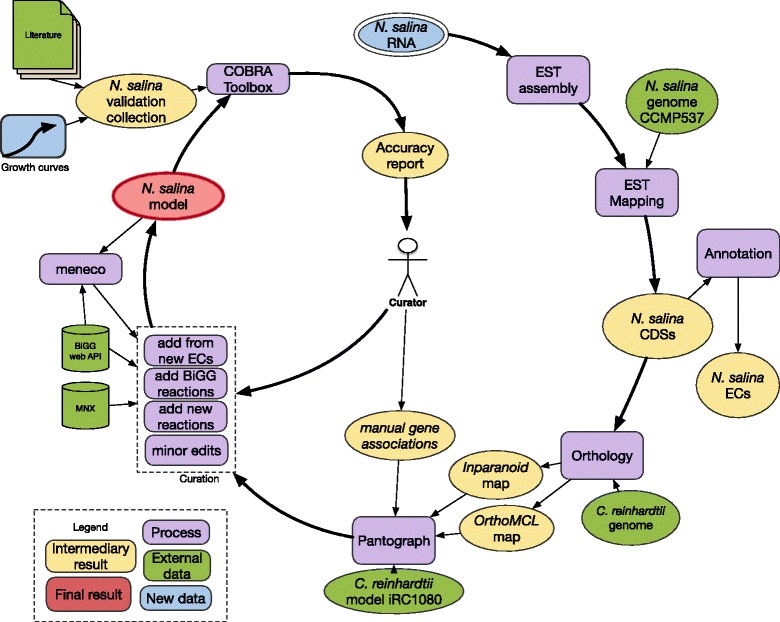
Reconstruction workflow for *N. salina* iNS934. We started with a collection of ESTs, that were mapped to an existing genome, finding new CDSs in the process. This set of new and old CDS was annotated and compared with *C. reinhardtii*, through Inparanoid and OrthoMCL. Then, using *Pantograph*, we projected the iRC1080 model to *N. salina*. This model was curated through a sequence of steps that added reactions from different sources. When the model was not functional, *meneco* provided hints about possible missing reactions. The resulting model was validated against a battery of 32 experimental observations. We used the COBRA Toolbox to manipulate and simulate models. The generated accuracy report guided our manual curators to edit the model, restarting the reconstruction process

We produced a version of our model in Systems Biology Markup Language (SBML) format in order to analyze it with compatible existing tools, and share it with the community. The model can be obtained as Additional file 3 and a diagram summarizing the reconstruction process is depicted in Fig. 1.

**Growth experiments for model validation** In order to validate our metabolic network, we prepared a battery of simulation tests based on 32 previously published growth experiments (see Table 1). These experiments comprise 23 cases in mixotrophic condition, 2 cases in heterotrophic condition and 1 in autotrophic condition. Additionally, 6 knockout experiments from Killian et al. (2011) were also included in this battery. We complemented this evidence by conducting experiments that could help us to improve and validate our model. We analyzed growth of *N. salina* in the presence of different nitrogen sources, phosphate and glucose. For experimental cultures we considered that growth was achieved if growth rate was greater than 1/3 of maximum growth among all experiments studied [66].

**Table 1:** Qualitative validation of iNS934

	Media condition	in vivo	in silico	Gene KO	TFPN	Reference	*Nannochloropsis* species
Luminosity	4 *μ*E (flourecent cool)	-	+		TN	[54]	sp
	40 *μ*E (flourecent cool)	-	+		FP	[54]	sp
	480 *μ*E (flourecent cool)	+	+		TP	[54]	sp
	5 *μ*E (flourecent warm)	+	+		TP	[91]	gaditana
	15 *μ*E (flourecent warm)	+	+		TP	[91]	gaditana
	50 *μ*E (flourecent warm)	+	+		TP	[91]	gaditana
	100 *μ*E (flourecent warm)	+	+		TP	[91]	gaditana
	200 *μ*E (flourecent warm)	+	+		TP	[91]	gaditana
	450 *μ*E (white led)	+	+		TP	[91]	gaditana
	1200 *μ*E (white led)	+	+		TP	[91]	gaditana
	2100 *μ*E (white led)	+	+		TP	[91]	gaditana
	3000 *μ*E (white led)	-	+		FP	[86]	sp
	Red led 673 nm, mixotrophic	+	+		TP	[92]	sp
	Red led 673 nm, autotrophic	+	+		TP	[92]	sp
Carbon	Glucose (mixotrophic)	+	+		TP	[93]	sp
	Glucose (heterotrophic)	+	+		TP	[93]	sp
	Ethanol (heterotrophic)	+	+		TP	[93]	sp
	Ethanol (mixotrophic)	+	+		TP	[93]	sp
	Inorganic carbon sources	+	+		FN	[94]	gaditana
Other	Sodium	+	+		TP	[95]	oculata
	Nitrite	+	+		TP	[96]	sp
	Phosphate	-	-		TP	This work	salina
	Nitrate	+	+		TP	[97]	This work
	Ammonium	+	+		TP	[98]	This work
	Sulfate	+	+		TP	[87]	gaditana
	Urea levels	+	+		TP	[99]	This work
Knockout	Ammonium	+	+	Nitrate reductase	TP	[96]	sp
	Nitrite	+	+	Nitrate reductase	TP	[96]	sp
	Nitrate	-	-	Nitrate reductase	TN	[96]	sp
	Ammonium	+	+	Nitrite reductase	TP	[96]	sp
	Nitrite	-	-	Nitrite reductase	TN	[96]	sp
	Nitrate	-	-	Nitrite reductase	TN	[96]	sp

For growth experiments conducted in our laboratory we used *N. salina* obtained from CSIRO (Commonwealth Scientific and Industrial Research Organisation). We used the complete f/2 medium as described by Guillard [55], which contained 75 mg/L of nitrate and 4.41 mg/L of phosphate. Experiments containing urea, nitrate and ammonium in the medium as the only nitrogen source were designed to have the same molar concentrations of nitrogen. Thus, we used a molar concentration of 8.8×10^−4^ for ammonium and nitrate and a molar concentration of 4.4×10^−4^ for urea. Before inoculation, cells were washed with ASW. We inoculated 1 L flasks with 2×10^6^ cells/mL, which were maintained at 25 °C, aireation of 0.5 VVM and to 60 *μ*mol m ^−2^ *s* ^−1^ of light intensity. For batch cultures growing with different carbon dioxide levels, it was supplied through the gas inlet in concentrations of 2 and 5%, respectively.

In order to diminish the internal reservoir of phosphate in cells for phosphate evaluation, inocula cells came from a culture with diminished phosphate concentration (0.4 mg/L phosphate). All cultures were followed by cell counting (Newbauer chamber and OD750 nm), biomass estimation (dry weight) and in some cultures nitrate consumption, evaluated using a microplate technique as described [67]. All experiments were performed in duplicate.

### Model analysis and validation

To analyze our iNS934 model, we used Flux Balance Analysis (FBA), Flux Variability Analysis (FVA) and dynamic Flux Balance Analysis (dFBA) from the Cobra Toolbox in MATLAB [68]. Flux Coupling Analysis (FCA) was performed using the F2C2 tool [69].

For model validation, we performed FBA with growth rate as the objective function to predict growth in different growth conditions mentioned in the previous section. For each test, we modified the flux boundaries of exchange reactions to simulate the composition of each growth media. We performed both a qualitative and a quantitative validation. Qualitative validation was used in cases where growth and/or uptake rates could not be obtained from the experimental data gathered from the literature and we only had growth/non-growth data. In these cases, we used an in silico scenario that simulates a rich medium by allowing free uptake of nutrients. The growth rate obtained in this condition was considered the reference maximal growth rate. For each experiment, we established that growth was achieved if the obtained growth rate was greater than 1/3 of the reference value [66, 70]. Then, we generated a confusion matrix and used the geometric mean as a measure of accuracy (See Table 1) to assess the predictive power of iNS934. To have a more accurate evaluation of our model predictions we performed quantitative validations by contrasting predicted growth rates with those obtained in in-house experiments. Prediction of growth rates was conducted using data from two sets of experiments. First, we used data from three batch cultures of *N. salina*: autotrophic growth with nitrate, ammonium and urea. Second, we used data from three batch cultures grown with nitrate at 0.03%, 2% and 5% of *CO*_2_ in the inflow gas. An in silico growth rate was obtained for each FBA simulation using fixed experimental uptake rates of nutrients and setting all other uptake reactions to zero. The error between experimental and predicted growth rates was then calculated.

We estimated uptake of *CO*_2_ by using the formula described previously [71]:

1 $$ CO_{2}biofixation= C*P*\left(MW_{CO_{2}}/MW_{C}\right).  $$

Values of biofixation were further transformed to specific uptake rates by dividing them by cell concentrations and the molecular weight of *CO*_2_. The specific uptake rate of *CO*_2_ used to perform simulations was calculated as the average specific uptake rate in exponential phase.

### In silico strain optimization

In order to find mutants of *N. salina* which may be useful for lipid overproduction, we developed a method for in silico strain optimization based on reaction knockouts. Our method guarantees the production of a target metabolism, while conserving the functional property, that is, the production of biomass. We iterated 200 times and sorted the resulting knockout sets. The metric to sort the results was defined as $m = \frac {\mu \times r_{t}}{|k|}$, where *μ* is the specific growth rate, *r*_*t*_ is the specific production rate of the target *t* and *k* is the knockout set.

This method consists of four steps: (1) We randomly traverse the reactions in the iNS934 model, removing reactions that are not needed for the production of the target metabolite. (2) Starting again from iNS934, we randomly traverse the model removing all reactions that are not needed for biomass production, while keeping those conserved in step 1. (3) Using FBA we check if the resulting model is able to produce the target metabolite when optimizing growth rate. If so, we continue to step 4. Otherwise we restart from step 1. (4) We try to recover the reactions removed during step 1 one at a time, checking that their inclusion maintains the integrity of the model to produce biomass and the target metabolite simultaneously. When a reaction breaks this restriction, it is included in the list of reactions to knockout.

## Results and discussion

### *N. salina* transcriptome mapping and annotation

**Fig. 2:**
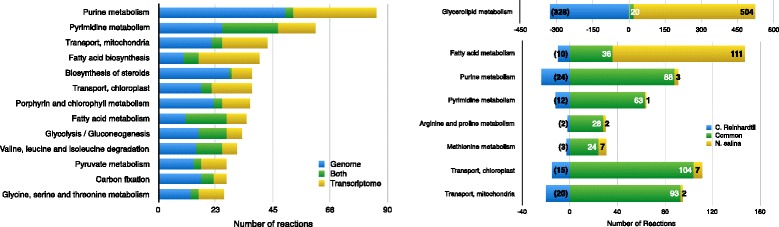
Contributions by Subsystem. *Left*: Number of *reactions* per subsystem in the model, with reactions projected using CCMP537 and from new CDSs. The new CDSs generated from our sequencing, complemented those obtained from the CCMP537 genome of *N. salina*. Several reactions were added based on these new CDS, adding new reactions to the model. In the graph, *blue* indicate the number of reactions with gene associations based on the CCMP537 genome, *green* when it depends on both, genome and our transcriptome, and *yellow* when only the transcriptome was used to support the existence of that reaction. Here we only show the thirteen subsystems with more reactions. *Right:* Exclusive reactions by subsystem. When comparing the models of *C. reinhardtii* iRC1080 and our *N. salina* iNS934, we can find reactions that are exclusive to iRC1080 (in *blue*), shared between both models (*green*) and exclusive to iNS934 (*yellow*). Here we show only selected subsystems *N. salina*, plus Glycerolipid metabolism, which was almost completely rewritten

We sequenced and assembled transcripts for *N. salina* and then combined them with the reported draft CCMP527 genome [58] to produce a comprehensive gene set for *N. salina*. From this set, 10913 putative genes identified in the transcripts of our *de novo* assembly were not contained in the reference genome (Additional file 5). These, together with the original genes in the genome, makes a total of 17519 putative genes. However, after the annotation, 7205 of these putative genes were not assigned a functional annotation and were therefore not considered in the metabolic reconstruction process. Out of the remaining genes, 3577 were assigned an EC number. In our metabolic model, 490 reactions were associated with putative genes from the transcriptome, which complemented the 1452 reactions predicted using only the CCMP527 genome. The contribution by subsystem of the iNS934 model is depicted in Fig. 2.

### iNS934: A genome-scale metabolic model for *Nannochloropsis*

We generated a functional GSMM, able to produce biomass, for the alga *N. salina*, called iNS934. The construction of our model started with an initial draft using reference iRC1080, a genome-scale model of *C. reinhardtii*. Even though models for other algæ have been built, as shown in Table 2, the level of detail varies among them. Only some of them are GSMMs including a genome-scale metabolic network reconstruction with more than a thousand reactions and a mathematical representation suitable for constraint based analysis. iRC1080 stands among them because it is a high quality model whose features include a carefully detailed light-driven algal metabolism, a multi-compartmentalized network and an extensive metabolism of lipids. Moreover, iRC1080 is part of the BIGG database and consequently, offers a controlled vocabulary of reactions and metabolites making it suitable for building GSMMs of standard language. Indeed, these features have been exploited before to construct other models of alga. For example, it was used as the template to build the models of *Chlorella vulgaris* [29], *Chrorella variabilis* [30] and *Phaeodactylum tricornutum* [35], and it was used in the gap-filling process for the model of *Emiliania huxleyi* [43].

**Table 2:** Metabolic reconstructions of algæ. List of metabolic reconstructions for algæ species

ID	Species	Genome-	Detailed glycero-	Multi-	Availability	in BiGG	Reference
		scale	lipid metabolism	compartment		database	
-	*C. protothecoides*	No	No	Yes	SBML	No	[31]
iCS843	*C. vulgaris*	Yes	Yes	Yes	SBML	No	[29]
iAJ526	*C. variabilis*	Yes	No	Yes	SBML	No	[30]
-	*C. pyrenoidosa*	No	No	No	No mathematical model	No	[33]
-	*C. sp FC2 IITG*	No	No	No	not available	No	[32]
EctoGEM	*E. siliculosus*	Yes	No	Yes	SBML	No	[42]
iEH410	*E. huxleyi*	Yes	No	Yes	SBML, MAT	No	[43]
-	*C. reinhardtii*	Yes	No	No	not available	No	[26]
iRC1080	*C. reinhardtii*	Yes	Yes	Yes	SBML, MAT	Yes	[22]
ChlamyCyc	*C. reinhardtii*	Yes	No	No	online	No	[27]
-	*C. reinhardtii*	No	No	Yes	XLS	No	[25]
-	*C. reinhardtii*	No	No	Yes	not available	No	[24]
iBD1106	*C. reinhardtii*	Yes	Yes	Yes	SBML	No	[21]
iCre1355	*C. reinhardtii*	Yes	Yes	Yes	SBML	No	[20]
AlgaGEM	*C. reinhardtii*	Yes	No	Yes	SBML	No	[23]
iLB1027	*P. tricornutum*	Yes	Yes	Yes	SBML	No	[35]
-	*P. tricornutum*	No	No	Yes	No mathematical model	No	[39]
DiatomCyc	*P. tricornutum*	Yes	No	No	Online	No	[40]
-	*P. tricornutum*	No	No	Yes	SBML	No	[41]
-	*P. tricornutum*	Yes	No	Yes	SBML	No	[38]
-	*P. tricornutum*	No	No	No	not available	No	[36]
-	*P. tricornutum*	No	Yes	No	No mathematical model	No	[37]
-	*O. lucimarinus*	Yes	No	No	SBML	No	[34]
-	*O. tauri*	Yes	No	No	SBML	No	[34]
-	*B. braunii*	No	No	No	No mathematical model	No	[28]

An orthology analysis revealed *C. reinhardtii* and *N. salina* shared 2612 orthologs, an amount that allowed us to obtain a reasonable initial draft to begin our reconstruction. This initial draft was improved and tailored to *Nannochloropsis* specific features using the annotation of the *Nannochloropsis* genome and transcriptome, and manual curation as described in materials and methods. An example of the contributions of manual curation to several subsystems can be seen in Fig. 2.

iNS934 describes 2345 reactions encoded by 934 genes, the 1985 metabolites consumed and produced by those reactions and a biomass function which describes the metabolic requirements for growth in autotrophic and mixotrophic conditions (see Table 3). From the total of reactions, 398 are transport reactions, 95 are exchanges with the media, 1613 are enzymatic reactions with a gene association and 239 without.

**Table 3:** Properties of genome-scale metabolic models

	*C. reinhardtii*	*N. salina*	*N. salina*
	(iRC1080)	(draft)	(curated)
Genes	1,1146	802	934
Reactions	2,191	1,897	2345
Metabolites	1,706	1,706	1985
Compartments	10	10	10

This table shows the properties of the template model (*C. reinhardtii*), the automatic initial draft produced by *Pantograph* and the results of the manual curation for *N. salina* iNS934

The model includes 10 different compartments: extracellular media, cytoplasm, mitochondria, chloroplast, thylakoid lumen, endoplasmic reticulum, peroxisome, Golgi apparatus, lysosome, and nucleus.

For the biomass function of our model, we started with the biomass functions of iRC1080. We adjusted these equations in order to represent the proportions of macro-nutrients found in *Nannochloropsis* species. In particular, we changed the coefficients related to DNA and RNA production according to previously described methodology [72]. The contribution of glycerolipids to biomass (Additional file 4) was obtained from a study carried out in *Nannochloropsis oceanica* by Li et al. [73].

It is well documented in several microalgæ that an increased lipid accumulation occurs under conditions where there is nitrogen starvation. In *Nannochloropsis* sp. growing in this condition, lipid production increases about one fold [74]. To simulate this behavior, we created a second biomass equation containing new stoichiometric coefficients based on proportions of glycerolipids of *N. oceanica* growing in a nitrogen-depleted condition (Additional file 4) [73].

In the following sections, we describe the main metabolic features and particularities of iNS934 with emphasis on lipid and nitrogen metabolism, key processes involved in production of targets with relevance in biodiesel and nutraceutical industries.

**Lipids**

The lipid metabolism in microalgæ is biotechnologically relevant, given that it is key for the production of biodiesel and food additives. Therefore the inclusion of an accurate and species-specific description of lipid pathways into a GSMM is needed if we want to use it as a platform to guide the production of these biotechnological targets. In order to describe the lipid metabolism of *Nannochloropsis*, we first added to iNS934 the biosynthesis pathways of polyunsaturated fatty acids (PUFAs) such as ETA(20:4), ARA(20:4) and EPA(20:5), which were absent in the *C. reinhardtii* model iRC1080 [73, 75]. This was a key step because PUFAs are relevant building blocks for glycerolipids in *Nannochloropsis*. Therefore their inclusion represents a major advance towards in silico simulation of lipid production in this algæ. Additionally, we also added unsaturated fatty acids, such as tetradecenoic acid (14:1) and hexadecadienoic acid (16:2), among others.

Once we added the pathways for all the required fatty acids, we replaced the glycerolipid pathways from the initial draft with 503 new *Nannochloropsis*-specific reactions that define the bioynthesis of TAG, diacylglycerol (DAG), phosphatidylcholine (PC), monogalactosyldiacylglycerol (MGDG), digalactosyldiacylglycerol (DGDG), sulfoquinovosyl diacylglycerol (SQDG), phosphatidylglycerol (PG), phosphatidylethanolamine (PE), phosphatidylinositol (PI) diacylglyceryl-O-4’-(N, N, N,-trimethyl) homoserine (DGTS) and free fatty acids. In particular, these 503 reactions account for specific proportions of glycerolipids into the biomass of *Nannochloropsis* and specific types of glycerolipids of *Nannochloropsis* with respect to other algæ. These glycerolipids could represent an important percentage of the *Nannochloropsis* biomass [75]. Given its importance, we created reactions to synthesize each of these glycerolipids and the corresponding stoichiometric coefficients required to account for the proportions in *Nannochloropsis*. The new reactions enable the use of iNS934 as a predictor of lipid metabolism in *Nannochloropsis*.

**Fig. 3:**
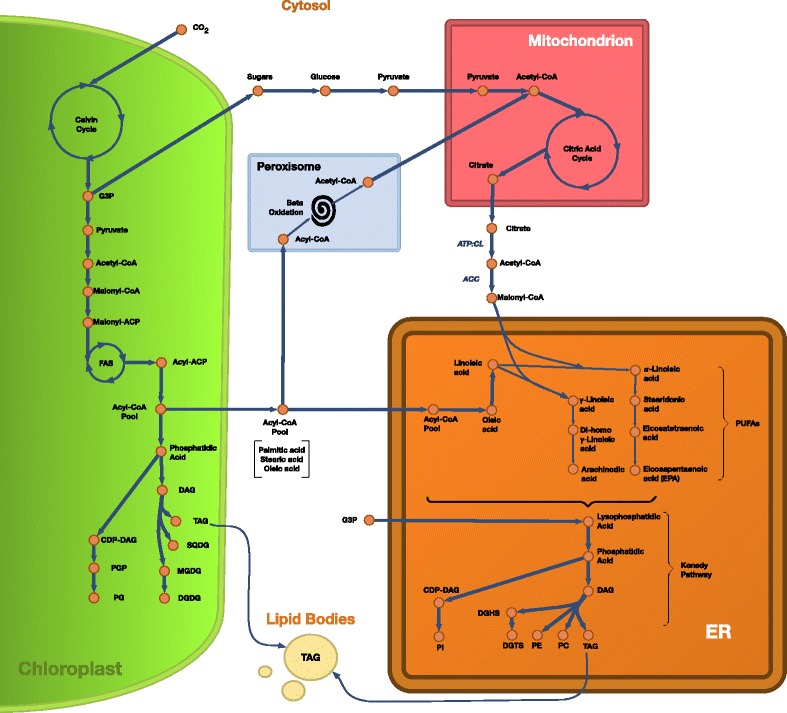
Diagram of lipid production in *N. salina*, as modeled in iNS934. Starting from CO_2_ we follow the chain of reactions until the production of lipids. The biosynthesis of lipids is performed at the Chloroplast and Endoplasmic Reticulum (ER) compartments. The Peroxisome contributes with the splitting of Acyl-CoAs into Acetyl-CoA, while the Mitochondria produce the citrate required Malonyl-CoA, key for the production of PUFAs at the ER. Most of the produced lipids end up in membranes (via the Biomass function), while TAG is also stored in lipid bodies. Inspired by diagrams from [46, 100] and [76]

Furthermore, based on current knowledge of species of chromista [76], we associated the biosynthesis of each glycerolipid to specific compartments, relocating associated metabolites and reactions. We added a compartment for the endoplasmic reticulum, where several glycerolipids are synthesized from fatty acids. Then, the biosynthesis pathways of PC, PE, DGTS and PI were located at the endoplasmic reticulum [77], while pathways for PG, DGDG, MGDG, SQDG were located at the chloroplast. Biosynthesis of TAG was located at both compartments. See Fig. 3 for a schema of our reconstructed lipid subsystem.

**Urea cycle**

**Fig. 4:**
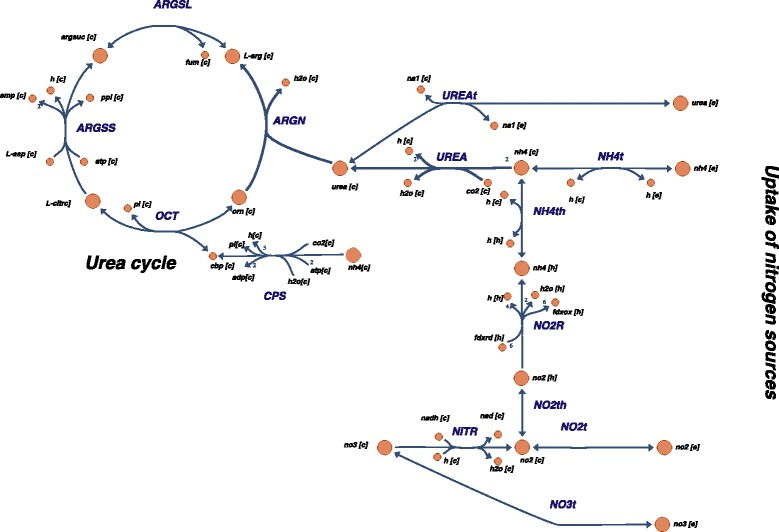
Nitrogen metabolism: Main reactions and pathways present in iNS934. ARGSL: Argininosuccinate lyase; ARGN:Arginase; ARGSS: Argininosuccinate synthase; CPS:Carbamoyl-phosphate synthase; OCT: Ornithine carbamoyltransferase; UREA: Urease; NO2R: Ammonia:ferredoxin oxidoreductase; NITR: Nitrate reductase; UREAt, NH4t, NO2t, NO3t : Urea, ammonia, nitrite and nitrate transport respectively

Nitrogen is quantitatively the most important nutrient affecting growth and lipid accumulation in various algæ [6]. In order to accurately represent the nitrogen metabolism in iNS934, we examined the reactions involved in this process. We found that *N. salina* has a complete urea cycle, including ornithine carbamoyltransferase, argininosuccinate synthase, argininosuccinate lyase, and arginase (Fig. 4). Additionally, we added transporters for nitrate, nitrite, ammonium and urea that have been identified previously in a *Nannochloropsis* genome [78]. Moreover we have determined experimentally that growth in *N. salina* can be sustained on nitrate, ammonium or urea as sole nitrogen sources (Additional file 5).

### *Overview of general properties of N. salina GSMM*

We performed three analyses in order to find the main network topological features describing the GSMM of *N. salina* iNS934. We started performing a FVA by maximizing and minimizing each reaction of the network. We compared this result with the ones obtained for the GSMMs of three other algæ, namely *C. reinhardtii* [22], *C. vulgaris* [29] and *P. tricornutum* [35]. We observed that curve shapes are similar between all networks. Moreover, we found that the amount of reactions having a narrow flux variation is similar: 174, 158, 150 and 379 reactions have a range of 0.01 mmol/gDW hr in *N. salina*, *C. reinhardtii*, *P. tricornutum* and *C. vulgaris* respectively. Additionally, we determined the number of blocked reactions in the network. We found that 38.7% of the reactions in *N. salina* were blocked, while this value decrease to 28.5% for *C. reinhardtii*, 11.9% for *P. tricornutum* and 38.2% for *C. vulgaris*. This result suggests that further refinement of the network is needed in order to accomplish a higher connection and dead-end elimination.

Second, we performed a FCA to determine coupled, partially coupled, directionally coupled and uncoupled reactions. We found that our model has almost the same proportions of types of reactions found in the compared algæ models. The uncoupled reactions far outnumber other types of reactions, representing nearly 90% of the total reaction pairs. Despite this, a higher percentage of fully and directionally coupled reactions can be observed in *N. salina* compared to the *C. reinhardtii* network, suggesting that the GSMM of *N. salina* is less connected.

Finally, we performed a connectivity and metabolite participation analysis. As is observed in other metabolic networks, we observed that a few metabolites participate in several reactions, meanwhile most metabolites participate in one or two reactions. As an example, we found usual currency metabolites such as H+, ATP, H_2_O, phosphate and coenzyme A between the 10 most connected metabolites.

### *Validation of N. salina GSMM*

To assess the predictive power of our iNS934 metabolic model, we simulated growth under different conditions using FBA and compared our results to experimental data. We reproduced existing observations of *Nannochloropsis* growth from the literature and from our experiments. Considering the lack of experimental evidence for *N. salina*, we based part of this validation on experiments from other *Nannochloropsis* species. We complemented this evidence by performing experiments that could help us improve and validate our model.

We modeled in silico autotrophic, mixotrophic and heterotophic media conditions by setting model constraints according to literature. In particular, for the autotrophic condition, we allowed the model to fix carbon from solar light measured from Earth’s surface. Carbon fixation through other types of light were set to zero. A maximum oxygen consumption uptake of 10 mmol/gDW was allowed. Water was allowed to travel only from the chloroplast to the cytosol and not viceversa. No carbon sources were allowed to enter the cell. Additionally, the chloroplastic enzymes divinylprotochlorophyllide vinyl-reductase, phosphofructokinase, glucose-6-phosphate-1-dehydrogenase and fructose-bisphosphate aldolase were turned off since these enzymes are inactivated with light [79, 80]. To model a mixotrophic condition, we applied the same restrictions except for carbon sources which were allowed to enter the cell. To simulate a heterotrophic condition, acetate, carbon dioxide and oxygen were allowed to enter the cell. Also a dark condition was modeled by inactivating light associated reactions.

Data on *Nannochloropsis* growth under different conditions gathered from the literature was not sufficient to calculate experimental growth and/or uptake rates in all cases, so we used these data for a qualitative validation, where only growth/non-growth prediction accuracy was evaluated. Qualitative assessment of metabolic reconstructions has been previously used to evaluate the biological capabilities of microorganisms on different experimental conditions. In particular, it has been widely used to study the consequences of environmental and genetic parameters that can be experimentally changed [81]. For example, this approach has been used to assess the prediction of nutrient requirements in different strains of *E. coli* [82] as well as other microorganisms [83] and to assess prediction of gene deletions in *S. cerevisiae* [84, 85]. Therefore, we carried out this simple analysis to explore a broader spectrum of scenarios for *Nannochloropsis* growth.

For a quantitative assessment of our model predictions we performed *N. salina* growth experiments using different nitrogen sources and different levels of *CO*_2_ and compared the obtained growth rates with those estimated in silico [85].

#### Qualitative validation

Table 1 shows the experimental conditions considered for qualitative validation. In all cases, biomass production both experimental and in silico were simplified into binary values (growth/no growth). Corresponding binary results obtained for all experiments were paired with simulations, with exact agreement in 29 cases (24 true positives and 5 true negatives). Three false positives were observed: *N. salina* growing at 3000 *μ*E *m* ^−2^ *s* ^−^1, 4 *μ*E and 40 *μ*E. In the first case an inhibition of growth was expected [86]. Unfortunately, GSMMs are not yet able to simulate inhibitions. Therefore, this behavior could not be reproduced. In the second and third case, it was expected that growth would be severely affected. However, growth rate was not decreased when compared with cultures grown under control conditions. This result is likely the product of over-optimistic flux simulations and can be reduced through parameter tuning.

The model predicted that phosphate was essential for growth. However, it has been shown that it is not needed to sustain growth experimentally [87]. Based on the model’s predictions, we decided to repeat experimentally the result reported in the literature. When we eliminated phosphate from the culture media we obtained growth in the first subculture. However, when we took cells from this first subculture without phosphate, new subcultures did not grow in a media without phosphate, but did grow in a media with phosphate. We presume that *N. salina* cells may accumulate phosphate granules as reservoirs which allowed them to grow in the first subculture and therefore the result reported by Forjan et al. [87] gave a false negative. In light of these new results, we concluded that phosphate was essential to sustain growth of *N. salina*. Therefore the prediction of iNS934 was considered accurate and was classified as a true negative result.

Overall, these growth/no growth simplified comparisons resulted in a prediction accuracy of 0.90 for the 32 evaluated conditions.

#### Quantitative validation

We assessed iNS934 quantitative predictive power by simulating *N. salina* growth in experimentally tested conditions. These conditions included growth on different nitrogen sources (nitrate, ammonium and urea) and different *CO*_2_ concentrations (0.03%, 2% and 5%) in the gas inlet. Measured uptake rates of nitrogen sources as well as estimated uptake rates of *CO*_2_ used as constraints in the model can be found in Additional file 6. Using these constraints we obtained an average error of 15% in prediction of growth rates (Table 4). This result indicates that iNS934 has a good level of accuracy since, in general, models are considered accurate when they achieve relative errors in growth rate predictions close to 10% [85, 88]. Further refinement of biomass composition as well as experimental measurement of *CO*_2_ uptake rate could improve growth rate predictions.

**Table 4:** Experimental and predicted growth rates of iNS934 Experimental and predicted growth rates for *N. salina* batch cultures growing in six different conditions

*CO*_2_	Nitrogen source	Experimental *μ*	Predicted *μ*	Error (%)
0.03%	Nitrate	0.0207	0.0169	22.1%
0.03%	Ammonium	0.0206	0.0156	32.2%
0.03%	Urea	0.0109	0.0098	11.1%
0.03%	Nitrate	0.0203	0.0186	9.3%
2%	Nitrate	0.0183	0.0207	11.8%
5%	Nitrate	0.0185	0.0178	4.2%

Nitrate, ammonium and urea were used independently as nitrogen sources and *CO*_2_ was used as the inorganic carbon source for each batch culture. Air (0.03% of *CO*_2_) and *CO*_2_ enriched air (2% and 5% of *CO*_2_) were used independently in the gas inlet

We also analyzed the inter-compartment fluxes of experimental conditions with different nitrogen sources in order to understand the main metabolic mechanisms that are involved in each case. In the first condition, the cell consumes nitrate as the nitrogen source. This is transformed to nitrite by the nitrate redutase in the cytosol. The nitrite is then transported to the chloroplast where it is further transformed to ammonium by the nitrite reductase. This ammonium is used to build some building blocks such as L-serine by the threonine ammonia-lyase (EC: 4.3.1.19). For carbon fixation, carbon dioxide entered the cytosol and was transported to the chloroplast where it participated in the Calvin Cycle. The malonyl-CoA used to build fatty acids is also created from carbon dioxide. Fatty acids such as decanoic acid and PUFAs such as eicosanopentanoic acid were synthesized in the chloroplast. PUFAs leave the chloroplast and travel to the endoplasmic reticulum to synthesize glycerolipids.

In the second condition, *N. salina* consumes ammonium which is transformed in the cytosol to urea (EC: 3.5.1.5) and amino acids such as L-glutamine, L-threonine and glycine. Urea enters the urea cycle and is further transformed to L-arginine. To simulate carbon fluxes, acetyl-CoA, which is synthesized in the cytosol, is transported to the mitochondria in order to generate energy and reducing power through the tricarboxylic acid cycle.

In the third condition *N. salina* consumes urea. The urea is transformed to ammonium by two consecutive reactions (EC: 6.3.4.6 and 3.5.1.54) in the cytosol. Additionally, ammonium is transported to the chloroplast where it is also used to synthesize glutamine and L-serine. The same mechanisms of carbon fixation and biosynthesis of fatty acids observed in experimental condition one was observed for cases where *N. salina* consumed ammonium or urea.

### Applications

#### Simulation of lipid production in nitrogen starvation

It has been shown that lipid content in *Nannochloropsis* changes when cells growing on a nitrogen replete media are transferred to a nitrogen-depleted condition. In particular, the content of TAG increases at least 100 fold [73]. We wanted to test if the iNS934 could predict this behavior at least qualitatively. To do this, using dFBA, we simulated cells growing in a batch culture that faced a sudden change in nitrogen availability. In this simulation we defined three stages. The first stage represented cells growing in a medium with a high availability of nitrate, used as the only nitrogen source. For this purpose we used the biomass equation in a nitrogen-replete condition and we set a maximum value for growth of 0.0045 h ^−1^ according to Simionato et al. [75]. At the end of the first stage, we simulated that cells were inoculated into a nitrogen-free media. This represented the beginning of stage 2. In this stage we changed the biomass equation to the one a for nitrogen-depleted condition and we set a maximum value for growth of 0.0036 h ^−1^ [75]. At the end of stage 2, cells were once again inoculated in a nitrogen-rich media. In stage three we again used the biomass equation for a nitrogen-replete condition.

**Fig. 5:**
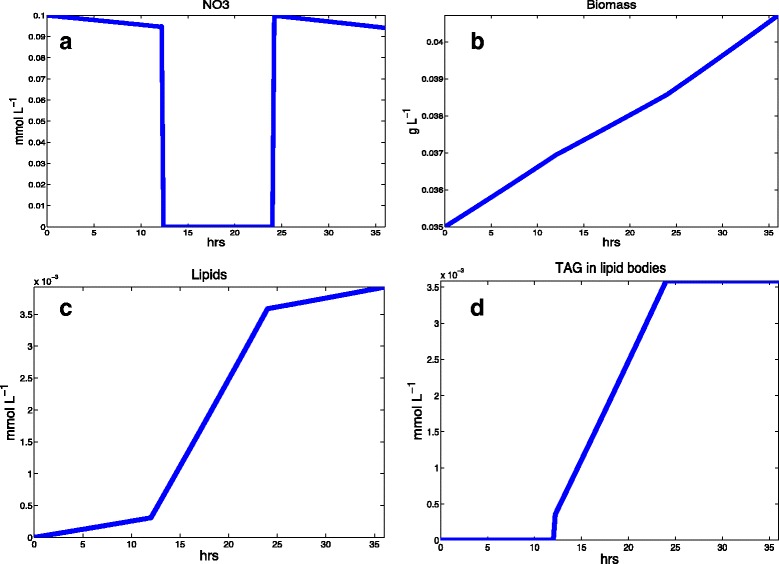
Dynamic FBA simulation of lipid accumulation in *N. salina* using iNS934. We simulated growth and lipid production using COBRA tools in three stages: NO_3_ available, NO_3_ depleted and NO_3_ available again. **a** NO_3_ available in the media. We set an initial amount of NO_3_ that was consumed by *N. salina* and eventually depleted. After this event, we added NO_3_ to the media, allowing *N. salina* to grow normally again. **b** Biomass accumulation of *N. salina*. After NO_3_ depletion, biomass production was reduced in stage 2 and increased again in stage 3. **c** Total lipid production (including TAG). In the first stage, lipid production increases according to the stoichiometric coefficients in the biomass formation reaction. Once NO_3_ is depleted, the lipid production increases in a higher rate than stage 1 due to the high amount of TAG produced toward lipid bodies. In stage 3, the lipid production rate is once again as described in stage 1. **d** TAG accumulated on lipid bodies. We created a compartment called “lipid bodies”, where the TAG produced, and not consumed by biomass, was stored

As shown in Fig. 5, in the first stage of our simulation, *N. salina* consumed nitrate and generated biomass according to the biomass equation for a nitrogen-replete condition. The glycerolipids were consequently increased as biomass increased. At the second stage, the lipid production increased significantly with respect to stage one. In the third stage, the growth rate and the lipid production were the same as stage one. This preliminary simulation showed that iNS934 is able to accurately describe the behavior in both nitrogen replete and nitrogen depleted conditions. Therefore this a feature that could be further exploited in biotechnological applications related to lipid optimization.

#### Using iNS934 to guide metabolic engineering of *N. salina*

As an example for iNS934 use, we focused on TAG production optimization as a case study. We used our model to search for sets of reactions whose blockage resulted in a higher *in silico* rate of TAG production in *N. salina*. This is a powerful and low-cost tool to predict the behavior of *N. salina* in different genetic and media contexts, working as a guide for metabolic engineering efforts. In order to find those in silico mutants, we initially used OptKnock [89] to obtain reactions whose group elimination allowed greater TAG production. However, we performed a FVA which revealed that removal of none of the reaction sets predicted by this tool guaranteed a minimum production of the desired lipids. We also tried OptForce [90], but we could not find reactions whose FVA indicated non-overlapping ranges of values. This is probably caused by the lack of experimental values that could constrain the possible fluxes of reactions in our model. Therefore, we developed an *in house* algorithm to determine sets of reactions that guarantee a minimum desired production, while keeping the capacity to produce biomass (see Methods). We applied this algorithm to optimize our target metabolites.

TAGs, like other glycerolipids, are represented as biomass constituents in GSMMs. This represents a challenge when optimizing these types of compounds because most strain design algorithms are conceived to optimize metabolic products which are secreted from the cell, such as succinate or lactate, not biomass constituents. To simulate an additional production of TAG, we created artificial reactions leading to the production of additional TAG. A demand reaction for that additional TAG was considered as the target objective function. Using our in-house algorithm for strain optimization, we found 82 sets of reactions whose independent elimination guaranteed a minimum TAG production (Additional file 7). We ranked those sets according to growth rate, minimum TAG guaranteed and number of knockouts. Higher growth rates and TAG productions as well as lower knockout sets lead to better scores.

The first observation we made is that growth rates for mutants were less than the growth obtained for wild-type. This observation makes sense since carbon must be redistributed into the biosynthesis pathways of lipids to produce more lipids, resulting in decreased carbon for use in other pathways. Since the carbon that was used to maximize growth rate in the wild-type is now being used for lipid biosynthesis in the mutant, the growth rate of the mutant must be less than the wild-type. The second observation we made is that, unlike wild-type, the additional TAG production is coupled with growth for all mutants. This explains why a minimum flux is guaranteed for the reaction producing additional TAG.

Most knockout sets involve reactions related to biosynthesis or transport of amino acids between different compartments. For example, one of these sets involves the knockout of a chloroplastic glutamate synthase, an enzyme that consumes glutamine and oxoglutarate to produce glutamate. The glutamate synthase knockout redirects the carbon flux that would be used to synthesize glutamate into the biosynthesis of fatty acids also located in this compartment. The forced flux into the biosynthesis of fatty acid leads to a forced flux into the biosynthesis of TAG. It is worth noting that glutamate is still produced in the cytosol satisfying the requirements for biomass production.

In the case of inter-compartment amino acid transport, the blockage of some transport reactions and the prevention of some transaminations in specific compartments, prevents the interchange of central metabolites, such as pyruvate and oxoglutarate, between compartments. This limits the paths in which they can be consumed, which redirects the carbon flux through triacylglicerol biosynthesis.

The results showed in this section demonstrate that iNS934 could be used to propose strategies for optimization of lipid production as well as other biotechnological targets. However, it is worth mentioning that these strategies only involve mass balances and other regulatory mechanisms are not considered. Further experimental information related to reaction knockouts could be used as input in the model to improve the identified strategies.

## Conclusions

We have reconstructed iNS934, the first genome-scale metabolic model of the marine algæ *N. salina*. To develop this model we used the alga genome annotation and additionally we generated transcriptomic data that allowed us to identify new putative genes for *N. salina*. iNS934 contains 1985 metabolites, 2345 reactions, 934 genes and 10 compartments, which in total, achieve a precise description of the metabolism of this alga. We tested iNS934 and we found it was able to make simple growth/non-growth predictions on 32 different conditions with an accuracy of 90%. These experiments included autotrophic, mixotrophic and heterotrophic conditions as well as key knockouts related to nitrogen metabolism. Moreover, a quantitative estimation of growth rates was achieved with an average error of only 15% for growth experiments with different nitrogen sources and *CO*_2_ supply levels.

iNS934 includes the *Nannochloropsis*-specific biosynthesis pathways for glycerolipids supported by experimental evidence. It has been shown that *Nannochloropsis* can grow to where 50% of its biomass is in the form of lipids. Thus, this model could be used to describe and predict the biosynthesis of lipids in high lipid producing species, especially for the biodiesel industry.

We employed iNS934 to find strategies for increasing the production of TAGs. We used a novel approach to handle the optimization of these biomass constituents, which could be used for other new strain optimization algorithms. Additionally, we created an algorithm of strain optimization which allowed us to find 82 sets of knockout reactions whose independent elimination in the network resulted in an improved production of TAG. The results highlight that further experimental information is needed in order to validate the knockout sets experimentally. The incorporation of regulatory mechanisms into GSMMs will probably allow users to predict strategies of strain optimization more accurately.

iNS934 could also be employed for other purposes such as metabolic engineering for improved production of omega-3 and omega-6 or improved production of beta-glucans. Both cases represent important cases of study for producing nutraceuticals with high value for human care.

## Endnotes

^1^ http://www.phrap.org/phredphrap/phrap.html

^2^ http://transdecoder.github.io

^3^ http://bigg.ucsd.edu/data_access

## Additional files

Additional file 1 CDS. New set of CDS of *N. salina* used for the reconstruction of iNS934. (FAA 6584 kb)

Additional file 2 Metabolites in iNS934. List of all metabolites in *N. salina* model iNS934. (XLSX 122 kb)

Additional file 3 Model. *N. salina* iNS934 model. SBML representation of the reconstructed model of *N. salina*. This file is compatible with SBML Level 3, Version 1, fbc ver. 2. It has been tested with COBRA Toolbox (2.0). (XML 4137 kb)

Additional file 4 Extra tables and figures. Tables and figures with details on model reconstruction and analysis. (XLSX 484 kb)

Additional file 5 ID mapping. Mapping of transcriptome of *N. salina* to CCMP537 genome assembly [58]. (CSV 1792 kb)

Additional file 6 Constraints used to predict growth rates. Measured uptake rates of nitrogen sources (Nitrate/ammonium/urea) and estimated *CO*_2_ uptake rates for batch cultures of *N. salina*. (XLSX 38 kb)

Additional file 7 Strain optimization. Proposed reaction knockouts for improving production of lipids in *N. salina*. (XLSX 107 kb)

## Abbreviations

ARA: Arachidonic acid
ASW: Artificial sea water
CDS: Coding sequence
CSIRO: Commonwealth Scientific and Industrial Research Organization
DAG: Diacylglycerol
dFBA: Dynamic flux balance analysis
DGDG: Digalactosyldiacylglycerol
DGTS: Diacylglyceryl-O-4’-(N, N, N,-trimethyl) homoserine
DIC: Dissolved inorganic carbon
DLC: Dark, Low CO_2_
EC: Enzyme commision
EPA: Eicosapentaenoic acid
ETA: Eicosatetraenoic acid
FBA: Flux balance analysis
FCA: Flux coupling analysis
FVA: Flux variability analysis
GSMM: Genome-scale metabolic model
HLHC: High light, High CO_2_
HLLC: High light, Low CO_2_
MGDG: Monogalactosyldiacylglycerol
PC: Phosphatidylcholine
PE: Posphatidylethanolamine
PG: Posphatidylglycerol
PI: Posphatidylinositol
PUFAs: Polyunsaturated fatty acids
SBML: Systems biology markup language
SQDG: Sulfoquinovosyl diacylglycerol
TAG: Triacylglycerol
